# The Development of an Evidence-Based Telephone-Coached Bibliotherapy Protocol for Improving Dementia Caregiving Appraisal

**DOI:** 10.3390/ijerph19148731

**Published:** 2022-07-18

**Authors:** Shanshan Wang, Daphne Sze Ki Cheung, Daniel Bressington, Yan Li, Angela Yee Man Leung

**Affiliations:** 1 School of Nursing, The Hong Kong Polytechnic University, Hung Hom, Kowloon, Hong Kong, China; shan-shan.wang@polyu.edu.hk (S.W.); yan-nursing.li@polyu.edu.hk (Y.L.); 2 WHO Collaborating Center for Community Health Services, The Hong Kong Polytechnic University, Hung Hom, Kowloon, Hong Kong, China; 3 School of Nursing and Health, Zhengzhou University, #101 Science Avenue, Zhengzhou 450001, China; 4 College of Nursing and Midwifery, Charles Darwin University, Casuarina 0815, Australia; daniel.bressington@cdu.edu.au

**Keywords:** bibliotherapy, dementia, caregiver, intervention, nursing

## Abstract

Caregiving appraisal is the caregivers’ cognitive evaluation of caregiving stressors. It determines the caregiving outcomes and caregiver health. Dementia caregivers have shown relatively negative caregiving appraisals. However, there is a lack of interventions to improve caregiving appraisal. This study describes the multi-phase process of developing and validating an evidence-based bibliotherapy protocol for improving the caregiving appraisal of informal caregivers of people with dementia. Two phases were included in the development: In Phase 1, a series of reviews of theory and evidence were conducted to identify the theoretical underpinnings, the core components, the dosage, and the mode of delivery of evidence-based bibliotherapy. In Phase 2, focus groups consisting of an expert panel of 16 clinicians and academics were used to validate the intervention protocol. Evidence synthesis was used in Phase 1 to formulate a draft intervention protocol. Content analysis was used in Phase 2 to work out the principles to revise the intervention protocol. The validated evidence-based bibliotherapy protocol included eight weekly sessions, and each session targeted improving one aspect of the essential factors that influence caregiving appraisal. This study provided a culturally sensitive and contextually appropriate evidence-based bibliotherapy protocol ready to be tested in a clinical trial.

## 1. Introduction

Caregiving appraisal is the cognitive evaluation of the potential stressor and the efficacy of one’s coping efforts [1]. According to Lawton’s two factor model, caregiving appraisal includes both the burdensome aspects of caregiving (i.e., caregiving burden and caregiving impact) as well as the rewarding aspects (i.e., caregiving satisfaction and caregiving mastery), which would lead to different well-being outcomes (i.e., depression and positive affect) for dementia caregivers [2]. Empirical studies found that caregiving appraisal “is also an important influencing factor of caregiver’s quality of life” [3]. A positive caregiving appraisal may also improve a care-recipient’s quality of life and prevent premature hospitalization [4]. Research found that dementia caregivers’ caregiving appraisal still needs improvement, but there is a lack of interventions [5]. The research team conducted a comprehensive review to identify the current services for dementia caregivers and the potential strategies that could be used among Chinese dementia caregivers. The review found that the community services for dementia caregivers were very limited, and due to the willingness of being filial to the family member with dementia, caregivers are inclined to seek help even though they perceive themselves to be exhausted in caregiving [6]. Under this circumstances, a culturally appropriate self-help intervention was determined as being the most viable option.

Self-help interventions have been conducted among dementia caregivers in several international settings, including the United States [7] and the Netherlands [8]. A published review reported that self-help interventions adopting cognitive behavioral therapy techniques could modify the caregivers’ thoughts and behaviors by facilitating self-help [9]. It de-emphasizes the therapeutic relationship between the interventionist and caregiver; even trained laypersons or paraprofessionals can use the intervention [9]. It is especially suitable for areas where professional resources are limited.

Bibliotherapy, a nonpharmacological intervention using reading materials to meet people’s therapeutic or developmental needs, is an innovative intervention as such [10]. Within bibliotherapy, participants are guided to self-identify and self-manage their problems through reading. Bibliotherapy was originally introduced to heal mental health problems, and gradually been used in other populations such as patients with cancer [11], caregivers of people with psychosis [12], adolescents [13], and even health professionals [14]. We did a systematic review of bibliotherapy and found that bibliotherapy could improve the depression, self-efficacy and state anxiety among informal caregivers of people with neurocognitive disorder (i.e., including dementia) [15]. It also has the potential to improve caregiving appraisal, because depression and self-efficacy are significantly associated with caregiving appraisal [16,17,18]. However, very limited bibliotherapy studies have been conducted among dementia caregivers, and no study has been conducted among Chinese caregivers; therefore, an evidence-based bibliotherapy protocol is needed [15].

Translating and adopting intervention manuals is a common practice in many experimental studies. However, as bibliotherapy requires the inclusion of real-life examples, manuals lacking the elements of Chinese culture may have limited use or relevance in Chinese populations. An original bibliotherapy manual is required for Chinese caregivers’ personal use and professional therapeutic practices, even though taking references from established manuals is appropriate. Hence, validation in the Chinese culture is needed when the existing evidence is from other cultures.

This study addressed the research need of improving caregiving appraisal by developing an evidence-based bibliotherapy protocol via a development-validation process. This novel intervention was developed to meet the needs of Chinese dementia caregivers, especially during the pandemic period, when group interventions are less feasible in implementation. The detailed reporting of the intervention development process will provide peer researchers insight into contextually appropriate intervention development and knowledge transfer.

## 2. Methods

### 2.1. Design

**Figure 1:**
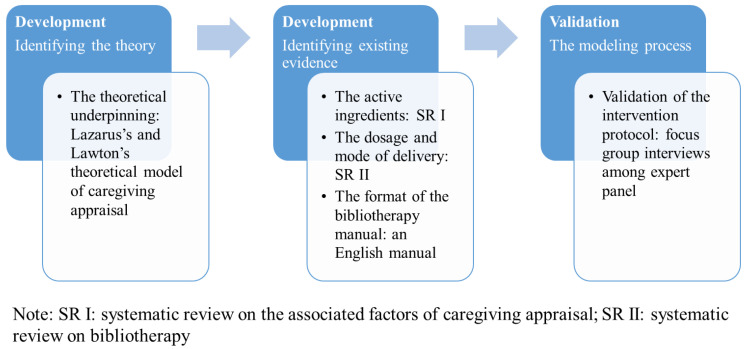
Methodological approach to developing an evidence-based bibliotherapy protocol.

Following the *MRC Framework for Developing and Evaluating Complex Interventions* (MRC Framework) [19], a multiple methods design was used to develop and validate the evidence-based bibliotherapy protocol. Figure 1 defines the theory and modeling phases of the MRC Framework and maps them onto the methods used to develop the bibliotherapy protocol. Triangulation of evidence (i.e., a series of reviews [6,15,18] and focus group interviews) was used to develop and validate the intervention.

### 2.2. Phase 1: The Intervention Development

#### 2.2.1. Identifying the Theory

Appraisal was firstly introduced in Lazarus’s *Stress*, *Appraisal*, *and Coping* theory [20]. In Lazarus’s theory, “cognitive appraisal” was utilized to represent a person’s cognitive evaluation of potential stressors. Stressor was the essential predictor of appraisal and initiated the appraisal process. Lazarus’s theory is the most classical theory regarding stress and appraisal, but it was developed for the general public. Lawton adopted Lazarus’s definition and developed a caregiving appraisal model for dementia caregivers. In this model, three factors were associated with caregiving appraisal: care recipient symptoms (the main stressor for the caregiver), caregiver health, and social support [2]. As both models are important for understanding caregiving appraisal, the current study used them as the theoretical underpinnings for intervention development. The theoretical relationship between associated factors and caregiving appraisal provided a rationale on the causal effect between the intervention components and outcomes of interest [21].

#### 2.2.2. Identifying Existing Evidence

Even though the two theoretical models provided evidence on the essential factors that would lead to the change of caregiving appraisal, updating the models was still deemed necessary because both of them were developed in the early 1990s. We synthesized the updated evidence by a systematic review of the associated factors of caregiving appraisal [18]. By synthesizing the updated associated factors and the factors in the theoretical models, the core components of the intervention were generated.

The dosage and mode of intervention delivery were identified from a systematic review on bibliotherapy [15]. In this systematic review, we found that the dosage of bibliotherapy usually included 3 to 12 weekly sessions, and the most commonly used mode of intervention delivery was via self-help manuals that were developed based on the caregivers’ therapeutic needs. Hence, this study proposed to use self-help manuals as the intervention delivery mode, and the dosage was determined to be one session per week. As the published bibliotherapy studies were not designed to improve caregiving appraisal, the contents of these interventions were not referenced.

The format of the bibliotherapy intervention manual was developed by referencing an English manual entitled “*The dementia caregiving skills program: reducing stress and enjoying time with your family member*” [21], because it has been well-tested among informal caregivers of people with dementia. Consent was provided by the original author to use their manual as a reference for developing our bibliotherapy manual. The first author, who has ten years of research experience in gerontological nursing and is experienced in editing caregiver textbooks, drafted the bibliotherapy protocol.

### 2.3. Phase 2: The Validation Process

Culture has been found to be an influencing factor of caregiving appraisal [18], and cultural mismatch may cause noncompliance or nonadherence to the intervention [22]. As the evidence from systematic reviews was identified from foreign studies, it was necessary to validate the intervention protocol among the target culture.

#### 2.3.1. Methods Used for Validation

Focus group interviews were conducted to validate the intervention protocol. Research shows that the responsibility of validation does not solely resides on the participant but involves all stakeholders [23]. The experts are essential stakeholders in dementia care, and they are the potential implementors of the intervention. Therefore, focus groups among experts (including academics and clinicians) were used to validate the intervention’s core components, dosage, and practicability. The validated intervention protocol was used among caregivers for feasibility and acceptability testing in the later stage of this research project and is published elsewhere [24].

#### 2.3.2. Inclusion Criteria of Participants

Experts from different backgrounds were interviewed to maximize the sample variation. The inclusion criteria of experts were: (1) nursing or medicine health care professionals who had at least a bachelor’s degree; (2) had at least five years working or research experience in gerontological nursing or gerontology; (3) had at least three years’ experience working with dementia caregivers.

#### 2.3.3. Sample

Purposive sampling was used to recruit participants. Four to eight participants were invited to each focus group. Experts with similar professional backgrounds but no power relationships were arranged into the same group to optimize participant engagement. Thematic saturation was used as the criteria for ceasing the interviews.

Three focus groups involving 16 experts were conducted. Each focus group lasted approximately one hour with a natural break. Most of the participants were female (93.8%). The average age of experts was 34.19 (SD = 5.96) years. The profession included academic staff (37.5%), nurses (43.8%), and doctors (18.8%). Two of the experts (12.5%) were also caregivers of a family member with dementia. Their working departments included neurology (37.5%), geriatrics (25%), and research centers (37.5%). Their education level included the doctoral level (6.3%), master’s level (56.3%), and bachelor’s level (37.5%). The average years of work in the gerontological area were 10.25 (SD = 6.97). The average years of work in dementia-related areas were 7.19 (SD = 5.65).

#### 2.3.4. The Interview Guide of the Focus Group

Open-ended questions were used for the interview, including “Overall, what do you think about the suggested sessions?”, “How may the information be suitable and useful for the caregivers you work with?”, “What do you think about the number of sessions and the length of each session?”, “What do you think about the sequence of the sessions”, and “What do you think should be added?”

#### 2.3.5. The Research Team and Reflexivity

For each focus group, there was a moderator and a notetaker. Both of them had qualitative research experience. The research team had no pre-existing relationships with the participants. Neither were they from the same institution. Participants were referred to the research team by their professional experience alone. During focus groups, the participants were introduced to the study objectives but no personal preferences from the interviewer. The focus groups were conducted in a meeting room that was neutral, comfortable, and accessible for participants. No observer was present apart from the moderator, note-taker, and the participants.

#### 2.3.6. Data Analysis and Trustworthiness

The audio recordings were transcribed within 24 h after the focus group and double-checked by academic staff. NVivo software version 12 was used for data management. Content analysis with an inductive approach was used to analyze data. The “Checklist for researchers attempting to improve the trustworthiness of a content analysis study” (Elo et al., 2014) was followed to ensure the trustworthiness of the content analysis. Two data analyzers independently worked on the coding, and a codebook was developed after they finished the first focus group. Member check was conducted to ensure credibility and confirmability. Principles for revision were extracted from the findings to guide the validation of the intervention protocol.

#### 2.3.7. Ethical Considerations

Ethical approval was obtained from the first author’s university. Principles of autonomy, nonmaleficence and beneficence, and confidentiality were followed. Written informed consent was received from each participant. Participation was entirely voluntary, and an identifier (i.e., a reference number) was given to each participant to ensure that no personal information was disclosed in the interview.

## 3. Results

### 3.1. The First Draft Intervention Protocol

#### 3.1.1. Core Components of the Intervention

Based on Lazarus’s and Lawton’s theoretical model and the findings of the systematic review [18], nine essential modifiable factors that would lead to the change of caregiving appraisal were determined to be the core components of the intervention (Supplementary Materials Table S1).

#### 3.1.2. Intervention Dosage and Mode of Delivery

Based on the number of core components, the dosage for the first draft intervention protocol was nine weekly sessions. Each week, strategies on changing one core component (in the form of a chapter) would be delivered to the participants. The mode of delivery was determined to be using an evidence-based self-help manual. However, weekly telephone coaching was also suggested to be essential for enhancing adherence [25]. A telephone coaching manual was therefore developed based on the caregivers’ manual, and structured questions were developed by referencing the English coaching manual. Hence, a draft bibliotherapy manual was developed for validation via focus groups (Supplementary Materials Table S1).

#### 3.1.3. Results of the Focus Group Interview

Four themes were generated from the content analysis: (1) Chinese culture and reading habits; (2) contents of the manual; (3) sequence of chapters; (4) dosage of the intervention. Corresponding principles of revision were developed based on the themes. Details of themes, categories, quotations, and principles of revision are in Supplementary Materials Table S2.

## 4. The Validated Intervention Protocol

The validated protocol included eight chapters. Each chapter covered a specific topic, and the sequence of chapters was re-arranged based on focus group findings. The caregivers would need to finish reading one chapter and receive telephone coaching each week (Table 1). The telephone coaching would be conducted by the interventionist following a coaching manual. Each coaching session included four sections: (a) greeting; (b) introducing the plan for this coaching; (c) monitoring, review, and problem-solving; (d) scheduling the next coaching. Troubleshooting plans for unexpected situations were also included in the coaching manual.

The duration of reading for each chapter depended on the caregiver’s pace, and the length of each chapter was ten pages on average. An orientation session was designed before the first session so that the caregivers know the aims, objectives and how to cooperation in each session. A booster session was developed in the fourth week to motivate caregivers’ involvement. A conclusion session was designed after they finished all the sessions.

## 5. Discussion

This paper describes the process of developing an evidence-based bibliotherapy protocol. Three significant steps were undertaken to develop and validate the intervention protocol to ensure cultural sensitivity and contextual appropriateness.

During the intervention development phase, evidence from different sources was used to ensure the rigor of design. As no bibliotherapy development paper has been published before, this study adopted a theory-based and evidence-based approach. The theoretical models explained the theoretical rationale that would lead to the change of caregiving appraisal. Evidence from the systematic reviews successfully facilitated the development of the intervention components, dosage, and mode of delivery. The multiple methods were in line with the intervention development approach that maximizes the future intervention effects [26]. The detailed reporting of the procedures ensured the rigor of the study and provided insight for researchers to assess the transferability.

There are similarities and differences between our newly developed intervention and the protocol we referenced [21]. Both studies used manual-based and telephone coaching-assisted intervention strategies and recognized the importance of stress management and behavioral problem management for dementia caregivers. Our intervention, however, included more diversified core components, covering factors from the social level (e.g., social support), interpersonal level (e.g., the dyadic relationship), and the individual level (e.g., self-efficacy). The reason was the difference in objectives. The referenced study was a stress reduction project, while our study aimed to improve the caregiving appraisal. Apart from stress, other factors that significantly influenced appraisal also could not be ignored such as family functioning and social support.

This study also highlighted the importance of validation when evidence was from a different culture. Culture and context are fundamental for successfully implementing a rigorously developed intervention protocol [27]. A unique aspect of this study was that we adopted a rigorous validation process, which provided valuable information to ensure successful implementation in the Chinese context. Although caregivers were not interviewed during the validation phase, experts’ opinions also guaranteed the information to be relevant and credible to the clients. In this study, the experts suggested using real-life examples in the Chinese culture, which are crucial for arousing participants’ psychological catharsis and insight. The expert comments would facilitate the feasibility of this intervention protocol and the acceptability for Chinese caregivers, which is published elsewhere [24].

This study contributes to the body of knowledge by proving an evidence-based bibliotherapy intervention for dementia caregivers. The process of intervention development and validation can provide peer researchers insight into intervention development, testing, and replication. It also contributes to new knowledge by developing an innovative, resource-saving, and easy-to-access evidence-based intervention that has great potential to solve trending issues. This study was designed to meet the specific needs of dementia caregivers who are in paradoxical situations of help-seeking caused by filial piety and social stigma. If demonstrated to be effective, the intervention protocol has the potential to be used by community health professionals.

Despite the strength of adopting strong evidence and multiple methods, this study still had some limitations. Although developers and stakeholders worked intensively during the intervention development process, only two of the stakeholders were caregivers. The caregivers, who were laymen, were not interviewed during the validation process because, in Chinese culture, it is less feasible to ask laypersons to comment on materials developed by professionals. This may have limited the potential feasibility of the intervention. A coproduction process including the end-users and stakeholders can be explored for future studies. Another limitation is the representativeness of the sample for the focus groups. Purposive sampling was used in this study. Because most of the professionals of the departments we contacted were female, especially nurses, the panel members involved in the focus group were mainly female, which may also have caused some bias. Future studies are suggested to consider the proportions in gender.

## 6. Conclusions

This is the first study to describe the development and validation of evidence-based bibliotherapy to support dementia caregivers. Multi-phase and multi-method approaches were used to develop an evidence-based intervention protocol, ready to be tested via clinical trials. This study is the first step in an interventional program that could ultimately improve dementia caregivers’ caregiving appraisal. The evidence-based principles underpinning this study could be transferable to evidence-based studies in nursing.

## Figures and Tables

**Table 1:** The validated evidence-based bibliotherapy protocol.

Weekly Tasks	Chapters	Main Components
Read Chapter 1 Telephone coaching	Chapter 1: Dementia and caregiver health	What is dementia Stages and symptoms of dementia Can dementia be cured How providing care can affect you as a caregiver
Read Chapter 2 Telephone coaching	Chapter 2: Care recipient behavioral problems	Learning more about behavioral problems Finding the “triggers” for problem behaviors Ways to change care recipient behavioral problems
Read Chapter 3 Telephone coaching	Chapter 3: Home safety and daily caregiving skills	How to ensure home safety How to deal with difficulties in daily care Some financial and legal issues in caregiving
Read Chapter 4 Telephone coaching	Chapter 4: Improving the caregiver and care recipient relationship	How to communicate with the care recipient Using nonverbal communication to improve the relationship Increasing pleasant events with the care recipient
Read Chapter 5 Telephone coaching	Chapter 5: Improving caregiving confidence	The importance of confidence in caregiving How to improve caregiving confidence Some “Basic Rights” of caregivers
Read Chapter 6 Telephone coaching	Chapter 6: Recognizing and relieving stress	Danger signals and how to recognize early signs of stress Skills of relaxation and why it is so important for caregivers Using relaxation in stressful caregiving situations
Read Chapter 7 Telephone coaching	Chapter 7: Depression in caregiving	Recognizing common symptoms of depression Depression and its effect on patients and caregivers How some little daily events can help prevent or reduce depression
Read Chapter 8 Telephone coaching	Chapter 8: Improving family coping and seeking social support	Family coping in dementia caregiving Ways to improve family coping How to seek help from relatives and friends How to seek help from professionals

## Data Availability

The data presented in this study are available upon reasonable request from the corresponding author.

## Supplementary Materials

The following supporting information can be downloaded at: https://www.mdpi.com/article/10.3390/ijerph19148731/s1. Table S1: The main components of the first draft evidence-based bibliotherapy protocol, Table S2: Themes, categories, quotations, and corresponding principles of revision generated from content analysis.
