# Supplementary material for: The Development of an Evidence-Based Telephone-Coached Bibliotherapy Protocol for Improving Dementia Caregiving Appraisal
Source: Int J Environ Res Public Health. 2022 Jul 18;19(14):8731. doi: 10.3390/ijerph19148731 (PMC9317862; doi:10.3390/ijerph19148731)
Supplement: Supplementary file 1 [file ijerph-19-08731-s001.zip › ijerph-1770115-supplementary.pdf]

**Supplementary Table S1** The main components of the first draft evidence-based bibliotherapy protocol

| Weekly tasks | Chapters | Main components of the chapter |
|---|---|---|
| Read Chapter 1<br>Telephone coaching | Chapter 1: Dementia and caregiver health | 1. What is dementia<br>2. Stages and symptoms of dementia<br>3. Can dementia be cured<br>4. The impact of caregiving on you as a caregiver |
| Read Chapter 2<br>Telephone coaching | Chapter 2: Care recipient behavioral problems | 1. Learning more about behavioral problems<br>2. Finding the "triggers" for problems in your caregiving situation<br>3. Developing an action plan to change behavior<br>4. How to handle problems in activities of daily living<br>5. Examples of managing specific problems |
| Read Chapter 3<br>Telephone coaching | Chapter 3: Social support | 1. What is social support<br>2. Why is social support important for caregiving<br>3. Ways to ask for help from others<br>4. Available social resources in community settings, and how to use social support |
| Read Chapter 4<br>Telephone coaching | Chapter 4: Self-efficacy | 1. What is self-efficacy<br>2. Self-efficacy in caregiving<br>3. Some "Basic Rights" of caregivers<br>4. Ways to enhance self-efficacy in caregiving |
| Read Chapter 5<br>Telephone coaching | Chapter 5: Family functioning | 1. What is family functioning<br>2. Importance of family functioning in caregiving<br>3. Ways of improving family functioning in caregiving<br>4. Communication tips with family and friends |
| Read Chapter 6<br>Telephone coaching | Chapter 6: Caregiver-care recipient relationship | 1. What is caregiver-care recipient relationship<br>2. Importance of caregiver and care recipient relationship in caregiving<br>3. Ways to improve caregiver and care recipient relationship<br>5. Learning activities for improving caregiver and care recipient relationship |
| Read Chapter 7<br>Telephone coaching | Chapter 7: Depression | 1. Depression and its effect on patients and caregivers<br>2. Some little daily events that can help reduce or prevent depression<br>3. Increasing pleasant daily events for you and your family member |
| Read Chapter 8<br>Telephone coaching | Chapter 8: Stress | 1. Danger signals and how to recognize early signs of stress<br>2. Skills of relaxation and why it is so important for caregivers |

| | | |
|---|---|---|
| | | 3. Using relaxation in stressful caregiving situations<br>4. Solving problems related to relaxation |
| Read Chapter 9<br>Telephone coaching | Chapter 9: Home safety and summary | 1. Home safety<br>2. Looking for the future: Financial and legal issues<br>3. Summary of the program |

**Supplementary Table S2** Themes, categories, quotations, and corresponding principles of revision generated from content analysis

| | | |
|---|---|---|
| **Theme 1: Chinese culture and reading habits** | | |
| *Categories* | *Quotations* | *Principles of revision* |
| 1a. Chinese culture | "Chinese have a different mode of thinking with the western people. The manual should be revised more fit the Chinese culture." (expert #6) "In Chinese culture, people would like to see the core problems." (expert #3) | Revise the format to be more culturally appropriate, such as problem-focused titles and subtitles, using easy-to-understand wording in Chinese daily life. |
| 1b. Chinese reading habits | "The Chinese like intuitive expressions, ……. For example, what is the problem, how do I solve the problem……" (expert #3)<br>"The formats of the manual should be more "problem-focused" so that it can follow the Chinese reading habit. For example, the titles can be changed to "how to solve the problem on …" (expert #5) | |
| **Theme 2: Contents of the manual** | | |
| *Categories* | *Quotations* | *Principles of revision* |
| 2a. Importance of daily caregiving skills | "Daily care is very important for caregivers. Behavior control, daily meals, changing clothes, home safety, anti-lost are all very important information for the caregivers (to know). They would like to learn more about this (area)……" (expert #2) | Add daily caregiving skills |
| 2b. Using culturally appropriate examples | "It's more realistic for the primary caregiver to seek help from relatives and friends. Nowadays, for people living in the city, even the neighbors may not be familiar with each other, it's not feasible to ask for help from neighbors or agencies" (expert #12)<br>Some of the examples are from western cultures, such as reading Bibles. However, most of the Chinese don't have religions. You may use the most common examples in their daily life, for example, trim vegetables……(expert # 7) | Use Chinese real-life examples in the manual |
| 2c. Avoiding terminologies | "The education level of caregivers should be taken into consideration. The words in the current version are a bit academic. The words should be as simple as possible. The simpler the phrase, the easier it is to be accepted" (expert #5) | Replace terminologies with daily expression |

| **Theme 3: Sequence of chapters** | | |
|---|---|---|
| *Categories* | *Quotations* | *Principles of revision* |
| 3a. Sequencing chapters based on caregivers' needs and concerns | "You should sequence the chapters in order of their concerns. Put what they care about most first so that they can read. There should be logic. For example, they should know what dementia is first, then what behavioral problem the patient have……then how to take care of the patient in daily life……then communication, then caregiver psychological aspects, for example, confidence, stress, depression…..." (expert #3) | Logically sequence the chapters based on caregivers' needs and concerns |
| 3b. Logical relations between chapters | "There should be logical relations between chapters, such as modular structure, so that participants won't feel jump thinking or getting lost" (expert #5) | |
| **Theme 4: Dosage of the intervention** | | |
| *Categories* | *Quotations* | *Principles of revision* |
| 4a. Dosage should be contracted | "Nine chapters shouldn't be a problem for young people, but it might be a little difficult for older people. You can condense the chapters to the most important parts……" (expert #9) | Condense dosage by combining content related chapters |
